# Service, Activism, and Friendships in High School: A Longitudinal Social Network Analysis of Peer Influence and Critical Beliefs

**DOI:** 10.1007/s10964-021-01549-2

**Published:** 2021-12-11

**Authors:** Christopher M. Wegemer

**Affiliations:** University of California Los Angeles, Los Angeles, CA USA

**Keywords:** Youth civic engagement, Sociopolitical development, Critical consciousness, Peer influence, Social network analysis, Marginalized youth

## Abstract

Scholars acknowledge that friends shape youth civic engagement, but the relative contribution of peer influence and critical beliefs to civic behaviors has yet to be disaggregated. Informed by sociopolitical development and critical consciousness theories, the present study used longitudinal social network analysis to examine peer socialization and adolescents’ awareness of systemic inequities in relation to participation in service and activist activities at a high school serving primarily low-income Latinx youth. Students were surveyed in May 2019 and May 2020 (*N* = 354; 51% female; in 2019, M_age_ = 15.9, age range 14.4 to 18.5). The results yielded evidence of peer influence on service activities, but not activism or perceptions of inequities. In contrast, adolescents’ perception of inequities predicted their activist behavior, but not service, after controlling for network effects and individual covariates. The school provided scaffolding for service activities, but not activist activities, potentially explaining the salience of service participation in youth friendship networks.

## Introduction

Adolescence is a critical period for sociopolitical development (Flanagan & Levine, 2010) and both socialization and individual beliefs can promote civic participation. Scholars have acknowledged the importance of peer influence on civic behaviors and beliefs during adolescence (Diemer & Li, 2011; Terriquez et al., 2020), yet the social processes that underlie youth civic engagement remain unclear. According to critical consciousness theory, adolescents’ awareness of systemic inequities is dependent on social interactions and reciprocally linked with civic action (Freire, 1970). Longitudinal studies that examine peer influence and perceptions of inequities are lacking, and further, methodologies that account for intertwined friendship formation and network processes are needed to avoid biased results (Sinclair, 2012). High schools provide opportunities for civic participation that simultaneously support adolescent friendships and exploration of critical perspectives (Watts & Flanagan, 2007). Explaining the relative contributions of peer influence and perceptions of inequities to sociopolitical development would have important implications for policies and practices in schools. The present study leverages longitudinal social network analysis to disentangle the extent to which peer influence and perceptions of inequities promote civic behavior at a local high school that primarily serves low-income Latinx youth.

### Peer Civic Socialization and Critical Consciousness

Youth are active agents in their own civic socialization (Flanagan, 2004) and adolescents tend to adopt the same political ideologies as their friends (Kandel, 1978). Youth can become more similar to their friends on civic behaviors and beliefs through a range of peer socialization processes. Behaviors and beliefs that are positively received by friends are mutually reinforced and more likely to surface again, whereas behaviors that are discouraged would be less likely to be repeated (Ryan, 2000). Adolescents have also been found to mimic the civic behavior of their friends and explicitly look to them as models (Gordon & Taft, 2011). Explicitly and implicitly, youth transmit information about social issues and opportunities to engage in civic activities through their friendship networks. For instance, political conversations among adolescents have been well-studied and research links the frequency of political discussions between friends to greater civic engagement (Diemer & Li, 2011). Peer influence can be conceptualized as the overall changes in behaviors and beliefs that result across peer socialization processes. The particular socialization mechanisms that manifest in the high school friendships of each adolescent are shaped by their particular sociocultural backgrounds and prevalence of oppression in their school community (García Coll et al., 1996).

Friendship networks provide a context for adolescents to encounter diversity and learn about experiences of oppression and privilege. According to critical consciousness theory (Freire, 1970), a reciprocal relationship exists between critical reflection and critical action, such that greater awareness of systemic inequities promotes civic behavior, leading to experiences that stimulate further reflection and action. The process through which youth develop perceptions of inequities is inherently interpersonal and requires consideration of social structures, interactions, and relationships (Watts & Hipolito-Delgado, 2015). Research suggests that strong peer relationships facilitate critical reflection (Landreman et al., 2007). The influence of peers may be particularly impactful for vulnerable adolescents who experience marginalization or navigate contexts where inequities are more pronounced. A recent study found that peer relationships bolstered critical perspectives and activist participation among Latinx adolescents who encountered oppressive circumstances (Terriquez et al., 2020).

Critical consciousness research suggests that Latinx adolescents’ awareness of systemic inequities may be an antecedent of involvement in activist activities that address the root causes of systemic social problems, but not service behaviors that preserve existing power structures (Diemer & Rapa, 2016). Accordingly, the present study differentiates between service and activist activities, aligned with sociopolitical development theory’s distinction between traditional civic participation and critical action (Watts & Flanagan, 2007). Youth may or may not participate in service with the intention of contributing to structural change, yet activism is explicitly and inherently critical, and the meaningful differences in antecedents and outcomes have been captured using similar multidimensional conceptualizations of civic participation (e.g., Westheimer & Kahne, 2004). Research on youth civic engagement has tended to focus on types of participation that do not challenge the status quo (Watts & Flanagan, 2007), which may overlook critical or culturally relevant activities and result in misleading conclusions regarding the civic engagement (or disengagement) of marginalized youth. Latinx adolescents may be more inclined to participate in critical civic actions that resonate with their direct experience of oppression (Suárez-Orozco et al., 2015). Compared to the intergenerational influence of family members, peer socialization may be more likely to encourage activism than traditional types of civic engagement (McDevitt & Kiousis, 2007).

Scholars have used network approaches to quantify socialization effects for a variety of adolescent beliefs and behaviors (Brechwald & Prinstein, 2011), yet literature remains sparse for youth civic engagement. The current study uses social network analyses to directly model peer influence, which advances research in the field in two ways. First, studies have historically employed surveys that ask youth to reflect and report on network-level processes or the behaviors of their peers. Although this approach may provide insight into adolescents’ perceptions of their peers’ civic engagement (and may be less burdensome on participants and researchers than capturing their friendship ties), youth do not have enough information to accurately evaluate network-level processes and their perceptions of their peers are often biased (Perry et al., 2018).

Second, studies of sociopolitical development typically use methods that assume independence of observations (such as linear regressions) and cannot adequately disentangle peer influence from other predictors and network processes (Sinclair, 2012). Similarities in peer groups can emerge over time through friendship formation and dissolution, which must be accounted to accurately estimate socialization and civic predictors (Veenstra & Steglich, 2012). Likewise, endogenous network effects may contribute to peer similarities, e.g., youth tend to reciprocate friendship nominations (*reciprocity*) and become friends with the friend of a friend (*transitivity*). In the present study, peer influence is modeled as the extent to which peers conform to their friends’ civic behaviors and perceptions of inequities over time, effectively capturing changes that result from socialization processes in the friendship network at a local high school.

### Opportunity Structures in School Contexts

Schools can support youth civic engagement through *opportunity structures* that facilitate access to civic activities, such as volunteer initiatives and activist afterschool clubs (Watts & Flanagan, 2007). These opportunities provide a crucial context for both political socialization and friendship formation, simultaneously shaping civic engagement and social networks. For example, a recent study examined school-based extracurricular activities using longitudinal social network analysis and found that friendship connections facilitated participation, and reciprocally, participation promoted the formation of new friendships (Schaefer et al., 2021). Research that centers the experiences of marginalized youth in school opportunity structures is needed, as studies of civic engagement tend to focus on dominant youth attending well-resourced schools (Watts & Flanagan, 2007).

The present study examines the coevolution of civic engagement and adolescent friendships at a public charter high school in southern California that provides students with regular opportunities to participate in volunteer activities (on and off campus) and robustly supports student government, but has neither student clubs that advocate for equity nor scaffolding that encourages participation in political movements, justice-oriented organizations, or critical campaigns. Generally, high schools tend to facilitate student involvement in service rather than activism and under-resourced schools typically provide fewer opportunities overall (Kahne & Middaugh, 2008). In the absence of school support for activism, friendships may play a relatively larger role in facilitating access to such activities. Research on social movements has documented ways that friendship ties can provide opportunities to participate in activist campaigns (Passy, 2003). Compared to school scaffolding, the degree to which youth social networks can effectively provide pathways to civic engagement is unclear. Relatedly, if a school does not prioritize critical perspectives in curricula, activities, or school culture, a student’s own critical beliefs may be a stronger predictor of their participation in activist activities independent of school structures or networks.

Adolescents’ development of civic engagement and critical consciousness is dependent on the culture of a school and its student body (Lenzi et al., 2014). The civic opportunity structures provided by a school may contribute to a school culture that renders certain civic behaviors and beliefs more salient to socialization and friendship formation than others. For instance, at a school that promotes service activities, participation in service activities may be valued among students and regarded as a socially desirable characteristic, and consequently, students who participate in service activities may be more likely to be selected as friends. Adolescents who do not embody valued characteristics may experience social marginalization, which can, in turn, facilitate the development of critical perspectives and action (Watts et al., 1999) or increase vulnerability to peer influence. Youth are typically more susceptible to peer influence at the beginning of high school compared to later in adolescence (Steinberg & Monahan, 2007) and tend to become more civically engaged as they matriculate through high school (Wray-Lake & Shubert, 2019). Both friendship networks and civic participation change from year to year and longitudinal approaches are needed to examine changes in social networks and civic engagement within the high school context.

## Current Study

Situated at the intersection of literatures on youth socialization and critical consciousness, the current study addresses gaps in understanding of peer influence and adolescents’ awareness of systemic inequities by employing longitudinal social network analyses. Two research aims guided the present study. The first research aim was to identify the extent to which friends influenced each other to adopt (or abandon) civic behaviors and beliefs over time. Students’ service, activism, and perceptions of inequities were anticipated to become more similar to the average level of their friends. Socialization effects were expected to be greater for service than activism and perceptions of inequities because the service opportunity structures provided by the school may make service more salient to friendships. The second research aim was to test the reciprocal relationship between critical reflection and action conceptualized by critical consciousness theory using analyses that accounted for social network processes. Adolescents’ perceptions of inequities were expected to predict increased participation in activism over time, and reciprocally, participation in activism was expected to predict increased perceptions of inequities. Relationships were not anticipated between perceptions of inequities and service behavior. Combined, the two research aims examine network effects and individual attributes as potential explanations of changes in youth civic engagement over time.

## Methods

### Participants

A survey was administered to all of the students enrolled at a high school in southern California at two time points. The surveys were conducted via an online platform during class time using a procedure that was compatible with constraints of the COVID-19 pandemic. In May 2019, 472 students completed the survey (91% of the school enrollment), and in May 2020, 435 students (84% of the school enrollment) completed the survey. Although the response rate was lower during the pandemic, survey completion remained consistent enough to satisfy the requirements of social network analyses. The final sample consisted of all students who were enrolled in the school in both years (*N* = 354), of which 272 (77%) completed the survey in both years. The majority of the participants were Latinx (85%) and low-income (70%). Between May 2019 and May 2020, 47 students (12% of those enrolled in May 2019) transferred to another school and were not included in the sample. Students who left the school were more likely to be male (*χ*^2^ = 4.85, *p* = 0.028) or White (*χ*^2^ = 5.38, *p* = 0.020), but did not differ on other demographic, educational, or civic characteristics. Descriptive statistics of the sample and attrition analyses are presented in Table 1.

**Table 1:** Descriptive statistics of the sample and attrition analyses

		All students enrolled in May 2019, excluding seniors	Final sample, students enrolled in both May 2019 and May 2020		
	Range	*N* (%) / M (SD)	*N* (%) / M (SD)	*χ*^2^/*t*	*p*
Female	0/1	197 (49.1%)	181 (51.1%)	4.85	0.028
Low-income status	0/1	284 (70.8%)	257 (72.6%)	1.26	0.262
GPA	0–4.83	3.00 (1.02)	3.15 (0.93)	1.46	0.146
Grade level					
Cohort graduating in 2022	0/1	148 (36.9%)	134 (37.9%)	0.02	0.890
Cohort graduating in 2021	0/1	137 (34.2%)	118 (33.3%)	0.93	0.335
Cohort graduating in 2020	0/1	116 (28.9%)	102 (28.8%)	1.16	0.282
Race/ethnicity					
Latinx	0/1	332 (83.0%)	299 (84.7%)	5.91	0.015
White	0/1	39 (9.8%)	30 (8.5%)	5.38	0.020
Asian	0/1	25 (6.3%)	21 (6.0%)	0.47	0.492
Black	0/1	4 (1.0%)	3 (0.9%)	0.69	0.394
Service behavior in May 2019	1–5	1.71 (0.93)	1.72 (0.93)	0.21	0.834
Activism behavior in May 2019	1–5	1.41 (0.79)	1.43 (0.79)	0.29	0.772
Perceptions of inequities in May 2019	1–6	3.36 (1.41)	3.39 (1.41)	0.28	0.778
*N*		401	354		

*Note*. Of the students enrolled at the school in May 2019, seniors (the cohort graduating in 2019) were excluded from the descriptive statistics because they could not possibly be enrolled in both years to participate in the study. (No seniors returned to repeat their senior year.) The final sample consists of all students who were enrolled in both May 2019 and May 2020. The table represents un-imputed data and some variables may contain missing values. Number and percent are shown for categorical variables, whereas mean and standard deviation are shown for continuous variables. Chi-square tests were applied to categorical variables. Fisher’s exact test was used for the indicator of Black students to accommodate low cell sizes. Unpaired *t*-tests were applied to continuous variables

### Measures

Civic behaviors and perceptions of inequities were assessed using items drawn from established inventories. All scales and subscales were validated by exploratory and confirmatory factor analyses that showed both divergent and convergent validity. Survey items are available in Appendix A.

#### Civic behaviors

Seven items were adapted from the youth civic engagement inventories of Corning and Myers (2002) and Diemer et al. (2017), based on Westheimer and Kahne’s (2004) typology of civic participation. Participants were asked how frequently they undertook a variety of activities on a 5-point Likert scale ranging from “Never did this” to “At least once a week.” The inventory was composed of two subscales. First, service behavior was captured with four items that assessed frequency of volunteering, organizing charitable events, attending religious groups, and participating in student government. Factor analyses indicated that one item (regarding attending religious groups) did not load adequately and was removed. The remaining three items were averaged together to produce a single indicator, which demonstrated acceptable reliability (*α* = 0.77 in spring 2019; *α* = 0.75 in spring 2020). Second, activist behavior was captured with three items that assessed frequency of participating in direct action, campaigning for issues, and involvement in social justice groups. The subscale demonstrated satisfactory reliability (*α* = 0.81 in spring 2019; *α* = 0.79 in spring 2020).

#### Perceptions of inequities

Four items measured perceptions of inequities along dimensions of race, class, gender, and sexual orientation, based on the critical reflection subscale of Diemer et al.’s (2017) critical consciousness inventory. These items assessed whether students believed members of certain racial/ethnic groups, people in poverty, women, or individuals who identified as gay or lesbian had fewer chances to “get ahead” in society, on a 6-point Likert scale from “Strongly Disagree” to “Strongly Agree.” The items demonstrated satisfactory reliability (*α* = 0.89 in spring 2019; *α* = 0.90 in spring 2020).

#### Friendship network

Each participant was asked to provide the first and last names of their five closest friends at the high school (consistent with common approaches to identify egocentric networks; Marsden, 2011). The names of their peers were linked with their respective survey responses.

#### Demographic and education-related indicators

Several indicators were constructed from high school record data obtained in spring 2019 and spring 2020 to serve as potential covariates. A dichotomous indicator was used to describe whether or not each participant was female. A nominal variable of race/ethnicity was based on four categories: Hispanic, White, Black, or Asian. A dichotomous indicator representing participants’ eligibility for free-and-reduced price lunch was used to capture low-income status (specifically, below 185% of the poverty line in either 2019 or 2020). School academic data was used to create a categorical variable of grade level in spring 2020. A continuous variable captured cumulative GPA on a 0–4 scale.

### Missing Data

Missingness of data ranged from 0% for demographic indicators and GPA variables to 16% for 2020 civic variables. (See Table 2 for the un-imputed sample sizes of each variable.) Single imputation was employed to account for missing data for all study participants. (Multiple imputation is generally preferable, but inappropriate for the present network analyses.) Separate imputation models were conducted for each of the two years. All study variables were included in the imputation models, and consistent with established practices, missing values were imputed using chained equations (see White et al., 2011). This approach allowed separate conditional distributions for each imputed variable, which was suitable for the present dataset, as several variables did not conform to normal distributions.

**Table 2:** Descriptive statistics and correlations among study variables

	1	2	3	4	5	6	7	8	9	10	11	12	13	14	15	16	17	18	19	20
1. Service behavior, 2019	1																			
2. Service behavior, 2020	0.53*	1																		
3. Justice behavior, 2019	0.57*	0.27*	1																	
4. Justice behavior, 2020	0.28*	0.42*	0.59*	1																
5. Perception of inequities, 2019	0.18*	0.04	0.23*	0.22*	1															
6. Perception of inequities, 2020	0.04	0.09	0.20*	0.24*	0.52*	1														
7. Female	0.01	0.09	0.09	0.12*	0.21*	0.26*	1													
8. Low-income status	−0.02	−0.09	0.07	−0.09	−0.08	−0.09	0.06	1												
9. GPA	0.12*	0.22*	−0.04	0.04	0.04	0.17*	0.11*	−0.12*	1											
10. Cohort graduating in 2022	−0.09	−0.12*	−0.09	−0.08	−0.15*	−0.01	0.03	0.02	0.17*	1										
11. Cohort graduating in 2021	0.02	0.07	0.04	0.05	0.02	−0.01	−0.02	0.04	−0.17*	−0.55*	1									
12. Cohort graduating in 2020	0.09	0.06	0.06	0.04	0.15*	0.02	−0.01	−0.06	−0.01	−0.50*	−0.45*	1								
13. Race/ethnicity: Latinx	−0.06	−0.09	0.08	0.03	−0.06	0.04	0.09	0.41*	−0.19*	0.01	0.00	−0.01	1							
14. Race/ethnicity: White	−0.05	0.01	−0.04	0.01	0.06	−0.06	−0.17*	−0.34*	0.05	−0.05	0.00	0.05	−0.72*	1						
15. Race/ethnicity: Asian	0.15*	0.13*	−0.05	−0.03	0.02	0.05	0.08	−0.23*	0.25*	0.08	−0.02	−0.06	−0.59*	−0.08	1					
16. Race/ethnicity: Black	−0.02	−0.04	−0.04	−0.04	0.00	−0.09	−0.03	−0.01	−0.06	−0.07	0.07	0.01	−0.22*	−0.03	−0.02	1				
17. Number of friendship nominations received, 2019	0.10	0.08	0.02	0.02	−0.08	0.08	0.04	−0.06	0.22*	0.07	−0.06	−0.01	−0.03	0.02	0.06	−0.12*	1			
18. Number of friendship nominations received, 2020	0.08	0.04	0.10	0.10	−0.04	0.14*	−0.02	−0.02	0.16*	0.05	−0.04	−0.01	0.05	−0.05	0.02	−0.06	0.52*	1		
19. Social isolate (no nominations), 2019	−0.01	0.04	0.06	0.07	0.01	−0.05	−0.10	0.03	−0.04	0.00	0.00	0.00	−0.04	0.00	−0.02	0.22*	−0.40*	−0.22*	1	
20. Social isolate (no nominations), 2020	0.00	0.03	−0.04	−0.04	−0.01	−0.07	0.06	−0.04	−0.11*	−0.04	0.04	0.00	−0.06	0.08	0.01	−0.03	−0.18*	−0.45*	0.12*	1
Mean	1.73	2.04	1.43	1.52	3.39	3.71	0.51	0.70	3.15	0.38	0.33	0.29	0.85	0.09	0.06	0.01	3.09	3.13	0.07	0.09
S.D.	0.93	1.08	0.79	0.81	1.41	1.41	0.50	0.46	0.93	0.49	0.47	0.45	0.36	0.28	0.24	0.09	2.11	2.16	0.25	0.28
Min	1	1	1	1	1	1	0	0	0	0	0	0	0	0	0	0	0	0	0	0
Max	5	5	5	5	6	6	1	1	4.83	1	1	1	1	1	1	1	15	13	1	1
*N*	328	297	327	297	322	297	354	354	354	354	354	354	354	354	354	354	325	289	325	289

*Note*. All variables are presented unstandardized and un-imputed. For dichotomous variables, the mean represents the proportion of participants

*p < 0.05

### Analytic Strategy

To investigate hypotheses regarding peer influence and perceptions of inequities, a stochastic actor-based model was estimated (SABM; see Snijders et al., 2010). The RSiena package (version 1.2–23, released January 12, 2020) was operated in R statistical software (Ripley et al., 2021). Survey responses from each student were used to construct a dataset representing the complete friendship network at the high school. Based on each student’s characteristics and the characteristics of their friends, longitudinal changes in civic attributes and friendships were simultaneously estimated by behavior and network functions (respectively).

First, the behavior functions estimated change and stability in civic outcomes (service, activism, and perception of inequities). Peer influence was captured by an effect that measured how likely students were to adopt civic characteristics closer to the average level of their friends (*average similarity* effect), after accounting for other network processes and individual attributes. Service, activism, and perception of inequities were modeled as potential reciprocal predictors of each other over time. The effect of popularity on civic attributes was measured as the extent to which students who were selected by a greater number of friends were more likely to have higher civic attributes over time (*indegree* effect). The function also included gender and grade level as potential covariates, as well as linear and quadratic terms that controlled for the distribution of each civic variable. The coefficients produced by the behavior function represented the change in log odds of increasing behavior one unit associated with a one unit change in the modeled characteristic.

The SABM algorithm required that the outcome variables (service, activism, and perceptions of inequities) consist of a small range of integers (Ripley et al., 2021). Accordingly, the variables were categorized to have values of 1, 2, or 3. Values were chosen to maximize conceptual similarities between the collapsed scale anchors and to maintain the distribution of each variable. For example, a score of 1 on the measure of service corresponded to the scale point “Never did this”, which represented the most common student response. A score of 2 represented moderate participation, “Once or twice last year” or “Once every few months.” A score of 3 represented frequent participation of “At least once a month” or “At least once a week.” A similar approach was used for activism and perceptions of inequities. All other variables in the model were mean-centered.

Second, the network function described the likelihood that within each dyad, a friendship tie would persist, dissolve, or that a new friendship tie would form across time. The modeled effects tested whether the characteristics of students (e.g., civic behavior, gender, etc.) were associated with friendship formation or dissolution. A positive coefficient for the ego effect would indicate that students with a particular characteristic were more likely to send friendship nominations over time, whereas a positive coefficient for the alter effect would mean that students with the same attribute were more likely to receive nominations (that is, they would become more socially desirable). A positive coefficient for the same/similarity effects would indicate that students had a tendency to become friends with peers who share a particular characteristic. The network function estimated selection effects for the primary civic variables of interest (service, activism, and perceptions of inequities) as well as controls for gender, race, grade level, and GPA. The coefficients of the network function represent the change in log odds that friendship ties will be added or persist (as opposed to not added or dropped) associated with a one unit change in a particular characteristic.

The network function also controlled for network features and endogenous processes that could influence friendship formation or dissolution and lead to biases in the effects of interest. Adolescents tend to reciprocate friendship nominations received from others, which is captured by a *reciprocity* effect. A transitivity effect controlled for the propensity of youth to become friends with the friends of a friend. Specifically, a *gwesp* term (geometrically weighted edgewise shared partners) was used to estimate the likelihood that two friends will have a friend in common over time, weighted to account for declining probability of adding subsequent shared friends. (A weight of log(2) is commonly used and produces good fit; Ripley et al., 2021). The model also included terms to assess the effects of popularity and isolation on friendship formation, specifically, the likelihood that students would receive more nominations in the future if an individual nominates more friends (*outPopSqrt*) and develop friendships in the future if an individual did not previously nominate any friends (*outIso*).

The model consisted of three behavior functions (for service, activism, and perceptions of inequities) as well as a network function for friendship ties. The estimation process involved a multitude of micro-steps in which each student was randomly selected and given an opportunity to change their friendships or behavior. The likelihood of change depended on the parameters specified in the model. The number of opportunities that each actor received was represented by the coefficient of the rate effect in each function. Consistent with best practices (Ripley et al., 2021), the SABM algorithm iteratively estimated the models in three phases, with six subphases in the second and 10,000 simulations in the third. Post hoc goodness of fit tests were conducted to validate model functionality and demonstrate the efficacy of the stochastic process in matching the characteristics of the network at the second time point (see Lospinoso & Snijders, 2019 for details). Prior to testing the model, correlational analyses and descriptive network statistics were evaluated to examine relationships between study variables and network features over time. The preliminary analyses were especially informative in light of the scarcity of literature describing youth civic engagement in social networks.

## Results

### Descriptive Statistics

Descriptive statistics and correlational analyses are presented in Tables 1 and 2, respectively. In 2019, 61% of students reported participating in service activities at least once a over the previous year compared to 70% of students in 2020, with a moderate correlation between the two time points (*r* = 0.53, *p* < 0.001). Similarly, in 2019, 52% of students reported participating in activist activities at least once a month compared to 46% of students in 2020, with a moderate correlation between the two time points (*r* = 0.59, *p* < 0.001). Mean levels of participation in activism were lower than service in both years (2019, *t* = 4.36, *p* < 0.001; 2020, *t* = 6.70, *p* < 0.001), although service and activism were moderately correlated in each of the waves (2019, *r* = 0.57, *p* < 0.001; 2020, *r* = 0.42, *p* < 0.001).

Descriptive statistics of the friendship network are presented in Table 3. The stability between the two waves of friendship networks (as measured by the Jaccard coefficient) was 0.29, sufficient for SABM analyses (Snijders et al., 2010) and aligned with other studies of adolescent friendship networks (e.g., Simpkins et al., 2013). Roughly half of the ties were reciprocated, also consistent with other studies of adolescent friendship networks (e.g., Block, 2015). Students had an average of about 6 friendship ties (the sum of both incoming and outgoing nominations) and a small number of student “isolates” had no ties (5 in 2019, 16 in 2020). Homophily, the tendency for friendship ties to exist between individuals with similar characteristics or behaviors, was evident in the network for all of the study constructs in both years, except students were more likely to be friends with others who had similar frequencies of participation in service and activism in 2020, but not in 2019.

**Table 3:** Descriptive network statistics

	2019	*p*	2020	*p*
Number of students who completed the survey	280		281	
Isolates	5		16	
Average outdegree	3.09		3.14	
Dyad census				
Mutual	235		273	
Asymmetric	625		565	
Null	61,621		61,643	
Edgewise reciprocity	0.43		0.49	
Transitive triads	851		980	
Density	0.009		0.009	
Homophily (Moran’s I)				
Service	0.05	0.204	0.26	<0.001
Activism	0.01	0.834	0.08	0.027
Perceptions of inequities	0.15	<0.001	0.16	<0.001
Female	0.50	<0.001	0.51	<0.001
Low-income status	0.22	<0.001	0.11	0.002
GPA	0.32	<0.001	0.28	<0.001
Race/ethnicity	0.25	<0.001	0.25	<0.001
Grade level	0.87	<0.001	0.82	<0.001

*Note*. The Jaccard coefficient, a measure of network stability across both waves, was 0.29. Outdegree is the number of friends nominated by each student. Isolates are students who did not receive or send any friendship nominations. Edgewise reciprocity is measured as the ratio of reciprocated edges to all edges. Density is measured as the ratio of observed ties to all possible ties, however, this only considers the number of nodes and does not account for the survey constraint of a maximum of five nominations per student. The Moran’s I indicator has a range of −1 to 1, with higher positive values indicating that students who are connected in the network are more similar on the particular attribute

### Longitudinal Model

A SABM was successfully estimated and optimized for best fit, exhibiting good overall maximum convergence t-ratio (0.08, acceptably below the 0.25 threshold). Additional diagnostic tests found satisfactory goodness of fit (see Appendix B). The model is presented in Table 4. The results addressed the two research aims regarding civic socialization and the reciprocal relationships between perceptions of beliefs and civic behavior, each described in turn below followed by findings about network selection, endogenous network effects, and individual-level covariates.

**Table 4:** SABM results, coevolution of friendships and civic behavior

	*b*	*SE*
Service behavior function		
Rate	1.56***	0.26
Linear shape	−0.44	0.35
Quadratic shape	0.47*	0.20
Service, average similarity	3.30**	1.25
Service, indegree	0.04	0.08
Effect from perception of inequities	−0.19	0.27
Effect from activism behavior	−0.22	0.46
Effect from 10th grade (12th is reference)	0.22	0.25
Effect from 11th grade (12th is reference)	0.08	0.26
Activism behavior function		
Rate	2.19***	0.45
Linear shape	−1.98***	0.47
Quadratic shape	0.35	0.30
Activism, average similarity	−0.39	1.16
Activism, indegree	0.01	0.09
Effect from perception of inequities	0.75*	0.38
Effect from service behavior	0.25	0.30
Effect from 10th grade (12th is reference)	0.25	0.29
Effect from 11th grade (12th is reference)	0.39	0.30
Perceptions of inequities behavior function		
Rate	1.69***	0.23
Linear shape	−0.37	0.34
Quadratic shape	−0.30	0.24
Perceptions of inequities, average similarity	1.44	1.13
Perceptions of inequities, indegree	0.21*	0.10
Effect from service behavior	−0.36	0.31
Effect from activism behavior	0.10	0.41
Effect from 10th grade (12th is reference)	0.07	0.25
Effect from 11th grade (12th is reference)	−0.21	0.25
Network function		
Rate	18.42***	1.52
Density (outdegree)	−2.50***	0.15
Reciprocity	2.60***	0.10
Transitivity (gwespFF)	1.78***	0.08
Popularity (outPopSqrt)	−0.26***	0.04
Isolates (outIso)	6.08***	0.43
Graduation year (same)	0.89***	0.07
Female, alter	−0.17**	0.06
Female, ego	0.03	0.13
Female, same	0.35***	0.06
GPA, alter	−0.06	0.03
GPA, ego	0.17*	0.07
GPA, similarity	0.61**	0.20
Race/ethnicity, same	0.21**	0.08
Service behavior, alter	0.31***	0.12
Service behavior, ego	0.16	0.18
Service behavior, similarity	0.72*	0.36
Activism behavior, alter	−0.32	0.50
Activism behavior, ego	−0.45	0.54
Activism behavior, similarity	−0.49	1.19
Perceptions of inequities, alter	0.05	0.08
Perceptions of inequities, ego	0.10	0.21
Perceptions of inequities, similarity	−0.28	0.33

*Note*. *N* = 354. The maximum convergence ratio was 0.08. For the gwespFF term, *α* = log(2)

*p < 0.05; **p < 0.01; ***p < 0.001

Regarding the first research aim, the results from the behavior functions provided evidence of civic socialization for service, but not for activism or perceptions of inequities. The average similarity coefficient in the service behavior function indicated that students tended to conform to the average level of their friends’ service behavior over time (*b* = 3.30, *p* = 0.008), above and beyond peer selection preferences accounted in the model. To interpret this effect, consider an example of an adolescent who initially did not participate in service activities (corresponding to a score of 1), but all of their friends participated at a moderate level (several times a year, corresponding to a score of 2). For each opportunity to change, the odds that this adolescent would increase their service behavior to participate at a moderate level are 5.21 times higher than remaining a nonparticipant (calculated as exp[3.30/2]). The linear and quadratic terms of each behavior function controlled for distributional changes in service, activism, and perceptions of inequities (specifically, tendencies towards higher or lower values, as well as regression to the mean or dispersion).

Regarding the second research aim, perceptions of inequities predicted increases in activism over time (*b* = 0.75, *p* = 0.049), after controlling for network and behavioral effects. However, activism did not reciprocally predict perceptions of inequities. The coefficient of perceptions inequities can be exponentiated to aid interpretation. The odds of a student increasing their frequency of participation in activism by one level (e.g., from not participating at all to participating several times a year) was 1.45 times higher for each additional level of perceptions of inequities that the student held (e.g., disagreeing that systemic inequities exist, having ambivalence about systemic inequities, or agreeing that systemic inequities exist). Perceptions of inequities did not predict service behavior (nor vice versa). Relatedly, service and activism did not longitudinally predict each other.

The SABM accounted for changes in friendships over time in order to avoid bias in the primary research aims. The network function indicated that service activities contributed to friendship formation, but activism and perceptions of inequities did not. Controlling for demographic factors and academic performance, students who had higher levels of participation in service activities were more attractive as friends to their peers (*alter* effect, *b* = 0.31, *p* = 0.009), but they were not more likely to nominate friends (*ego* effect, *b* = 0.16, *p* = 0.356). Students were more likely to form friendships with others who had similar levels of participation in service activities (*similarity* effect, *b* = 0.72, *p* = 0.044). The alter, ego, and similarity effects are best interpreted in tandem by calculating odds ratios to represent the likelihood of friend selection based on the service behavior. Specifically, for a student who did not participate in service activities, the odds of selecting a friend who also did not participate were 0.10 higher than the odds of selecting a friend who participated at least once a month. In contrast, for a student who participated at least once a month in service activities, the odds of selecting a friend who participated similarly were 1.03 higher than the odds of selecting a friend who did not participate. The effects of popularity on civic outcomes (conceptually distinct from friend selection preferences modeled by the network function) were estimated by the *indegree* terms of the behavior functions. The more popular a student was (the more friendship nominations they received), the more likely they were to increase their perceptions of inequities over time (*b* = 0.21, *p* = 0.044). Popularity was not related to service and activism behavior.

As anticipated, the controls of the network function were statistically significant. Across the school network, students demonstrated a strong tendency to reciprocate friendship nominations (*reciprocity*, *b* = 2.60, *p* < 0.001), to prefer friends of their friends (transitivity measured as *gwespFF*, *b* = 1.78, *p* < 0.001), and to select friends if they did not nominate any in the previous year (*outIso*, *b* = 6.08, *p* < 0.001). Students who nominated a high number of friends were less attractive as friends to others (*outPopSqrt*, *b* = −0.26, *p* < 0.001). The density (*outdegree*) effect serves as the equivalent of an intercept in the model and controls for the total number of friendship ties in the model, with negative values indicating that fewer ties are present than would be expected by chance.

The model controlled for several individual-level attributes that yielded statistically significant effects. Friendships tended to form and endure between peers who were similar in grade level (*b* = 0.89, *p* < 0.001), gender (*b* = 0.35, *p* < 0.001), race/ethnicity (*b* = 0.21, *p* = 0.009), and GPA (*b* = 0.61, *p* = 0.002). Females were less likely to be nominated as friends than males (*alter* effect, *b* = −0.17, *p* = 0.005) and students with higher GPAs selected more friends compared to students with lower GPAs (*ego* effect, *b* = 0.17, *p* = 0.011). After accounting for network and behavior effects, grade level was not a statistically significant predictor of service, activism, or perceptions of inequities.

An alternative model was estimated as a robustness check (see Table 5 in Appendix C). Existing literature suggests that peer influence may depend on both the strength of the relationship between friends and each friend’s popularity (Brown & Larson, 2009). Accordingly, instead of simply estimating the tendency of students to conform to the average level of their peers, the alternative model tested a different effect (*avSimRecPop*) that examined the extent to which the adolescents adopted the civic attributes of the subset of their friends with whom they had a reciprocated relationship, weighted by their friends’ popularity (measured as the totally number of friendship nominations each friend received). The model yielded results that were consistent with the original model; peer influence was evident for service behavior (*b* = 0.38, *p* = 0.029) and perceptions of inequities predicted activist behavior (*b* = 0.76, *p* = 0.046).

## Discussion

Despite the importance of peers in adolescent sociopolitical development, few studies have investigated civic socialization using social network techniques. Similarly, the reciprocal relationship between activism and adolescents’ awareness of systemic inequities is central to critical consciousness theory, yet longitudinal studies that examine critical beliefs and their social underpinnings are lacking. Increased understanding of the social mechanisms underlying civic engagement could inform impactful practices and policies in school contexts, particularly for marginalized youth. The present study leveraged a cutting-edge methodological strategy to accomplish the study’s aims regarding peer influence and reciprocal links between perceptions of inequities and civic behavior. Processes of socialization and friendship selection were present for service activities, but not activism, whereas perceptions of inequities positively predicted later activism, but not service.

Addressing the first research aim, the model provided evidence of peer influence on service behavior. Specifically, students tended to adopt or abandon service behaviors to conform to the average level of participation of their friends over time. By directly modeling peer influence using longitudinal social network analyses that attribute agency to adolescents, the findings build on previous youth civic socialization literature (Flanagan, 2004). To accurately estimate peer influence, friendship formation and dissolution was also accounted for. Students displayed a preference for peers who had equal levels of participation in service activities, with stronger effects for those who participated more frequently. The findings suggest that similarities between friends on service behavior emerge through both peer socialization and selection processes at the school, also providing a potential explanation for the emergence of political homophily detected in recent studies of adolescent friendship networks (Oosterhoff et al., 2021).

In contrast to service behavior, neither peer influence nor friendship selection effects were observed for activism or perceptions of inequities. The discrepancies may be due to the school’s frequent coordination of volunteer opportunities and active support of student government. Co-participation in school-supported activities provides a foundation for socialization and changes in friendships through semi-structured peer interactions in a shared space (Schaefer et al., 2021). The absence of opportunity structures for activism potentially rendered critical activities less relevant in peer interactions. Interestingly, homophily was present for perceptions of inequities in both years, and popular students were more likely to increase their perceptions of inequities, yet neither of the network features were explained by socialization and selection processes. The effects may be accounted for by other network processes (e.g., byproducts of curricular sorting at the school or socialization effects from friendships beyond the school context), which would be important to investigate in future research.

As anticipated, perceptions of inequities predicted increases in activism over time but not service, addressing the second research aim. The distinction between activism and service emerged despite a moderate correlation between the two types of civic behavior. The findings extend critical consciousness research that found perceptions of inequities predicted protest behavior for Latinx youth, but not voting or conventional political behavior (Diemer & Rapa, 2016). Contrary to the notion of praxis in critical consciousness theory (Freire, 1970), activism did not reciprocally predict perceptions of inequities. Several explanations are possible. Changes in an adolescent’s perceptions of inequities were accounted for by popularity or other network effects, which may have otherwise been attributed to participating in activism. Also, research has found activism to be differentially linked to subscales of critical reflection (Diemer et al., 2017); activism may be reciprocally related to critical beliefs other than perceptions of inequities. Lastly, activism may not have been prevalent enough among the student body for the model to detect a predictive association from activism to perceptions of inequities. More research is needed to understand the breadth of social processes and critical beliefs that underlie potential reciprocal links between critical reflection and action in the high school context.

Overall, the findings have two potential implications for school policies and practices. First, the school’s scaffolding for service did not appear to promote students’ involvement in other civic activities more broadly, as service and activism were not predictive of each other. Further, peer socialization and friendship formation may not compensate for the absence of activist opportunity structures at the school, as the results did not provide evidence of social pathways for adopting activist behaviors. Second, the observed socialization and friendship formation processes were aligned with the school’s civic infrastructure, which suggests that school support for civic activities could shape the social and political environment of the student body. Generally, schools employ extracurricular opportunity structures and progressive curricula to foster civic engagement, but greater attention to implications of student social networks in civic behaviors and critical beliefs could lead to more effective policies and practices. The high school in the study is likely similar to most others in its support for service rather than activism, but because the present study centered on one high school and did not explicitly assess student co-participation in the same activities, it was limited in its ability to draw conclusions about school infrastructure. Future work will use measures and analytical techniques that more robustly capture the intersection of youth friendship networks and school opportunity structures (and potentially critical curricula) among diverse adolescents across multiple high schools.

In response to the continued call for greater representation of marginalized youth in civic engagement literature (e.g., Anyiwo et al., 2020), the current study foregrounds the predominately low-income Latinx student body served by the high school. The social networks of students appeared to facilitate engagement in service activities, which suggests civic initiatives that center agentic relational decisions of youth could represent asset-based alternatives to interventions that frame marginalized students as passive or disengaged. Relatedly, youth voice could be integrated into empirical studies to gain greater understanding of students’ lived experiences and support more specific claims about how social dynamics facilitate youth civic engagement and how critical consciousness may function differently for marginalized and dominant youth (see Wray-Lake & Abrams, 2020 for an example). Future work should extend the findings of the present study by combining social network analysis with mixed-methods approaches at a school with a heterogenous student body to examine demographic differences in potential links between school culture and peer socialization in specific activities beyond the limited dichotomy of service and activism. In such a study, simultaneous examination of critical beliefs and activist behavior could clarify existing research that has found critical activities may be more salient for youth of color than dominant youth (e.g., Suárez-Orozco et al., 2015) and advance understanding of the different types of critical beliefs that motivate civic behavior among diverse adolescents (Wegemer, 2021).

The second wave of the study was conducted during the COVID-19 pandemic, a unique historical moment that represents both an opportunity and a limitation. Students’ self-reported participation in service activities was higher during the pandemic compared to the year before (*t* = 4.34, *p* < 0.001) and there was no statistically significant difference in activism. Although the reasons for changes in service are unclear, it is possible that students may have responded to the public health crisis by contributing to service initiatives that alleviated the COVID-19 burden on others. This would be consistent with studies that reflect a sense of social responsibility among youth for practicing protective social distancing (Oosterhoff et al., 2020). Relatedly, the increased salience of civic activities during the pandemic may partially explain why homophily was present for service and activism in May 2020, but not May 2019; clustering among students may have been determined by a wider array of factors that eclipsed civic behavior during in-person classes compared to online classes. The network effects suggested that the pandemic may have shaped students’ friendships (e.g., the number of isolates increased from 2019 to 2020). The Black Lives Matter protests that followed the murder of George Floyd in summer 2020 and the contentious presidential election in fall 2020 both occurred after the current study was conducted. Follow-up studies will investigate subsequent changes in service and activism. More broadly, further research will be important to understand the ways in which the unprecedented historical circumstances may have shaped the sociopolitical development of the current generation of adolescents.

## Conclusion

Extensive research has suggested that peers are important for political socialization and critical consciousness, but studies that disentangle the social mechanisms underlying youth civic development are sparse. Social network approaches are capable of providing novel insight into adolescent sociopolitical development. The present study found evidence of overlapping processes of socialization and friendship selection, but only for service behavior that was associated with school infrastructure. The results also add nuance to critical consciousness theory’s notion of praxis between activism and adolescents’ awareness of systemic inequities by highlighting the need to account for social dynamics. Longitudinal stochastic actor-based modeling holds substantial potential for not only advancing understanding of youth civic engagement, but also for informing tailored interventions in policies and practices that equitably support participation. School administrators who recognize that peers are effective socializing agents may prioritize accessible spaces and semi-structured opportunities for engaging in critical civic activities and ideas, which could profoundly shape the sociopolitical development of all youth in the broader friendship network. Future research will continue to highlight the civic experiences of marginalized youth and clarify social pathways to civic engagement.

## Figures and Tables

**Fig. 1:**
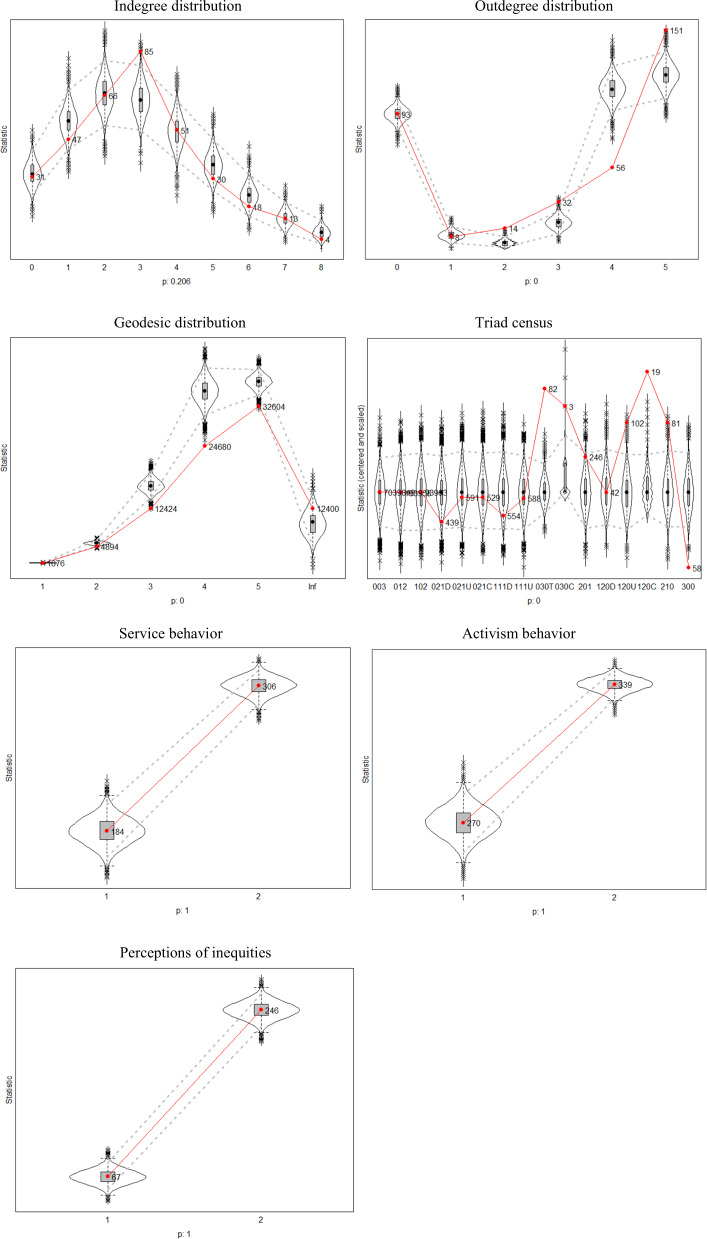
Goodness of fit statistics for the SABM model represented in Table 4

## Data Availability

The data collected and analyzed in the current study are not publicly available, but are available from the author on reasonable request.

## Appendix A

Survey inventories

*Civic behavior*

Over the last year, how often have you done any of the following activities?

[Responses on a 1–5 Likert scale: “Never did this,” “Once or twice last year,” “Once every few months,” “At least once a month,” or “At least once a week.”]

Participated in student government

Participated in a religious group (besides attending church)

Volunteered for [BLINDED HIGH SCHOOL NAME] or any organization (above and beyond the volunteer hours required for school)

Helped organize a food drive, fundraiser, or community event (at school or for another organization)

Signed an online or written petition about a social or political issue

Participated in a group that advocates for human rights, gay rights, women’s rights, or immigration rights

Joined in a protest march, political demonstration, or political meeting

*Perceptions of inequities*

In our society…

[Responses on a 1–6 Likert scale: “Strongly Disagree,” “Mostly Disagree,” “Slightly Disagree,” “Slightly Agree,” “Mostly Agree,” or “Strongly Agree.”]

Certain racial or ethnic groups have fewer chances to get ahead.

Poor people have fewer chances to get ahead.

Women have fewer chances to get ahead.

People who are gay or lesbian have fewer chances to get ahead.

*Friendship network*

Think about your closest friends from school. Write up to five names on the lines below, starting with your closest friend first. Please include their first and last name. Try to spell them as best as you can.

Remember, your responses will be kept confidential. Your survey will NOT be connected to your name and your responses will NOT be shared with anyone. Please complete this question as well as you can. [Five open-ended responses including first name, last name, and grade level]

## Appendix B

Goodness of fit tests for SABM analyses

The goodness of fit of the SABM described in Table 4 was assessed by evaluating the extent that the simulated networks had similar characteristics as the observed data. Statistics for the outdegree distribution, indegree distribution, geodesic distance, triad census, and behavior distributions (corresponding to service, activism, and perceptions of inequities) are shown in Fig. 1. The red lines indicate the statistics of the observed data. The majority of the measurement classes fell within the 95% confidence intervals generated by the SABM, shown in the panels in Fig. 1. The fit of the outdegree distribution was constrained by survey item that ascertained friendship nominations, which limited responses to five friends. The parameters of the SABM were iteratively adjusted to optimize fit without overspecifying the model. See Ripley et al., 2021 for more information regarding SABM goodness of fit tests.

## Appendix C

Results from alternative analyses

**Table 5:** Alternative SABM results, coevolution of friendships and civic behavior

	*b*	*SE*
Service behavior function		
Rate	1.53***	0.26
Linear shape	0.60*	0.30
Quadratic shape	0.32	0.18
Service, average similarity X recip X pop	0.38*	0.18
Service, indegree	0.04	0.07
Effect from perception of inequities	−0.20	0.24
Effect from activism behavior	−0.20	0.40
Effect from 10th grade (12th is reference)	0.28	0.23
Effect from 11th grade (12th is reference)	0.15	0.23
Activism behavior function		
Rate	2.20***	0.46
Linear shape	−1.91***	0.43
Quadratic shape	0.37	0.29
Activism, average similarity X recip X pop	−0.01	0.20
Activism, indegree	−0.01	0.10
Effect from perception of inequities	0.76*	0.38
Effect from service behavior	0.25	0.30
Effect from 10th grade (12th is reference)	0.23	0.29
Effect from 11th grade (12th is reference)	0.37	0.28
Perceptions of inequities behavior function		
Rate	1.70***	0.22
Linear shape	−0.35	0.33
Quadratic shape	−0.39	0.20
Perceptions of inequities, average similarity X recip X pop	0.34	0.21
Perceptions of inequities, indegree	0.21*	0.10
Effect from service behavior	−0.37	0.29
Effect from activism behavior	0.12	0.41
Effect from 10th grade (12th is reference)	0.07	0.24
Effect from 11th grade (12th is reference)	−0.21	0.26
Network function		
Rate	18.33***	1.22
Density (outdegree)	−2.50***	0.14
Reciprocity	2.60***	0.10
Transitivity (gwespFF)	1.79***	0.07
Popularity (outPopSqrt)	−0.26***	0.04
Isolates (outIso)	6.07***	0.37
Graduation year (same)	0.90***	0.07
Female, alter	−0.17**	0.06
Female, ego	0.03	0.13
Female, same	0.36***	0.06
GPA, alter	−0.06	0.03
GPA, ego	0.17*	0.07
GPA, similarity	0.61**	0.20
Race/ethnicity, same	0.21**	0.08
Service behavior, alter	0.29**	0.10
Service behavior, ego	0.18	0.19
Service behavior, similarity	0.71*	0.29
Activism behavior, alter	−0.33	0.37
Activism behavior, ego	0.47	0.50
Activism behavior, similarity	−0.50	0.94
Perceptions of inequities, alter	0.05	0.08
Perceptions of inequities, ego	0.10	0.18
Perceptions of inequities, similarity	−0.28	0.29

*Note*. *N* = 354. The maximum convergence ratio was 0.09. For the gwespFF term, *α* = log(2)

**p* < 0.05; ***p* < 0.01; ****p* < 0.001
